# Phylogenomics of fescue grass-derived fungal endophytes based on selected nuclear genes and the mitochondrial gene complement

**DOI:** 10.1186/1471-2148-13-270

**Published:** 2013-12-12

**Authors:** Piyumi N Ekanayake, Maia Rabinovich, Kathryn M Guthridge, German C Spangenberg, John W Forster, Timothy I Sawbridge

**Affiliations:** 1 Department of Environment and Primary Industries, Biosciences Research Division, AgriBio, the Centre for AgriBioscience, 5 Ring Road, Bundoora, Victoria 3086, Australia; 2 Dairy Futures Cooperative Research Centre, Bundoora, Australia; 3 La Trobe University, Bundoora, Victoria 3086, Australia

**Keywords:** Festuca, Pasture grass, Whole genome sequencing, PerA, TefA, Tub2

## Abstract

**Background:**

Tall fescue and meadow fescue are important as temperate pasture grasses, forming mutualistic associations with asexual *Neotyphodium* endophytes. The most frequently identified endophyte of Continental allohexaploid tall fescue is *Neotyphodium coenophialum*, while representatives of two other taxa (*Fa*TG-2 and *Fa*TG-3) have been described as colonising decaploid and Mediterranean hexaploid tall fescue, respectively. In addition, a recent study identified two other putatively novel endophyte taxa from Mediterranean hexaploid and decaploid tall fescue accessions, which were designated as uncharacterised *Neotyphodium* species (UNS) and *Fa*TG-3-like respectively. In contrast, diploid meadow fescue mainly forms associations with the endophyte taxon *Neotyphodium uncinatum*, although a second endophyte taxon, termed *N. siegelii*, has also been described.

**Results:**

Multiple copies of the translation elongation factor 1-a (*tefA*) and β-tubulin (*tub2*) ‘house-keeping’ genes, as well as the endophyte-specific *perA* gene, were identified for each fescue-derived endophyte taxon from whole genome sequence data. The assembled gene sequences were used to reconstruct evolutionary relationships between the heteroploid fescue-derived endophytes and putative ancestral sub-genomes derived from known sexual *Epichloë* species. In addition to the nuclear genome-derived genes, the complete mitochondrial genome (mt genome) sequence was obtained for each of the sequenced endophyte, and phylogenetic relationships between the mt genome protein coding gene complements were also reconstructed.

**Conclusions:**

Complex and highly reticulated evolutionary relationships between *Epichloë-Neotyphodium* endophytes have been predicted on the basis of multiple nuclear genes and entire mitochondrial protein-coding gene complements, derived from independent assembly of whole genome sequence reads. The results are consistent with previous studies while also providing novel phylogenetic insights, particularly through inclusion of data from the endophyte lineage-specific gene, as well as affording evidence for the origin of cytoplasmic genomes. In particular, the results obtained from the present study imply the possible occurrence of at least two distinct *E. typhina* progenitors for heteropoid taxa, as well the ancestral contribution of an endophyte species distinct from (although related to) contemporary *E. baconii* to the extant hybrid species*.* Furthermore, the present study confirmed the distinct taxonomic status of the newly identified fescue endophyte taxa, *Fa*TG-3-like and UNS, which are consequently proposed to be renamed *Fa*TG4 and *Fa*TG5, respectively.

## Background

*Neotyphodium* endophytes are asexual fungal species that form mutualistic interactions with a number of cool-season grasses, including ryegrasses (*Lolium* spp.) and fescues (*Festuca* spp.). Endophytes are disseminated through dispersal of plant seeds and obtain nutrition and protection from the host plant, while conferring superior persistence characteristics on the grass, such as improvements of mineral uptake and drought tolerance [1,2]. Furthermore, symbiotic fungal endophytes provide protection from vertebrate and invertebrate herbivores to the host plant through production of bioprotective alkaloids. To date, four major classes of alkaloids have been identified from endophyte infection of host grasses [3]: peramine and lolines, which deter invertebrate predation [4-6], and indol-diterpenes and ergot alkaloids, which are toxic to grazing vertebrates such as ruminant livestock [7,8]. Tall fescue (*Festuca arundinacea* Schreb. syn. *Lolium arundinaceum* [Schreb.] Darbysh.) and meadow fescue (*Festuca pratensis* Huds. [syn. *Lolium pratense* (Huds.) Darbysh.]) are two fescue taxa that are particularly important as temperate pasture grasses, and form associations with *Neotyphodium* endophytes. Tall fescue exhibits multiple ploidy level variants from tetraploid to decaploid [9,10]. Furthermore, within the hexaploid type, the commonly cultivated Continental and Mediterranean morphotypes have been deduced to arise from differing diploid progenitor genomes [11]. The most frequently identified endophyte of Continental allohexaploid tall fescue is *Neotyphodium coenophialum* (Morgan-Jones et Gams) Glenn, Bacon et Hanlin [12], while representatives of two other taxa, *Festuca arundinacea* taxonomic group 2 (*Fa*TG-2) and *Festuca arundinacea* taxonomic group 3 (*Fa*TG-3), have been described as colonising decaploid and Mediterranean hexaploid tall fescue, respectively [9,13]. In addition, a recent study based on simple sequence repeat (SSR) genotyping identified two other putatively novel endophyte taxa from Mediterranean hexaploid and decaploid tall fescue accessions, which were designated as uncharacterised *Neotyphodium* species (UNS) and *Fa*TG-3-like [9] (later named as *Fa*TG-4:[14]) respectively. In contrast, diploid meadow fescue mainly forms associations with the endophyte taxon *Neotyphodium uncinatum* (Gains, Petrini and Schmidt) Glenn, Bacon, Price and Hanli, although a second endophyte taxon, termed *N. siegelii*, has also been described [15].

All of these previously characterised and novel endophyte taxa of tall and meadow fescue exhibit heteroploid genome constitutions, based either on presence of multiple copies of known gene sequences [14,16-18] or generation of multiple PCR amplicons from specific SSR loci [9,19]. On this basis, such endophytes have been proposed to originate from sexual *Epichloë* species through a series of interspecific hybridisation events. Although the probable origins of several *Neotyphodium* species are believed to be well understood, those of the novel UNS and *Fa*TG-3-like taxa are yet to be determined. In addition, the degree of resolution of such phylogenomic analysis is a function of both the number and nature of the DNA sequences used to perform such studies.

Nuclear protein-coding gene sequences have been employed in previous studies of fungal endophytes to elucidate evolutionary relationships at the intraspecific and interspecific levels, through phylogenetic analysis of partial sequences representing orthologous intronic regions of the translation elongation factor 1-a (*tefA*), β-tubulin (*tub2*) and actin (*act1*) genes [14,18,20,21]. However, each of these genes encodes proteins that control essential functions in eukaryotic genomes, and are hence not exclusive to either fungal species or, indeed, *Neotyphodium* endophytes. Phylogenetic analysis of gene sequences that are specific to the *Epichloë*-*Neotyphodium* lineage could hence provide higher resolution of the phylogenetic affinities between sexual and asexual endophyte taxa. The *perA* gene catalyses synthesis of the invertebrate deterrent alkaloid peramine [6], and hence provides an ideal candidate. In previous studies, the number of multiple *perA* gene copies present in heteroploid endophytes has been shown to be consistent with proposed hybrid origins, irrespective of peramine production levels [6]. In addition, as a presumably dispensable gene, *perA* gene exhibits a higher rate of molecular evolution than the essential *tefA* and *tub2* genes [22], providing the capacity to resolve close taxonomic relationships. In a previous study, phylogenetic analysis based on sequenced PCR amplicons from the *perA* gene was performed for a selected set of fescue-derived endophytes [23]. However, inclusion of a larger number of additional *perA* genes, including those from putative progenitor *Epichloë* species, would be expected to improve the resolution of analysis and obtain a deeper understanding of *Epichloë*-*Neotyphodium* phylogenomics.

In addition to nuclear genes, sequence variation within the mitochondrial (mt) genome may be used to fully interpret the interspecific hybridisation process by which heteroploid endophytes are believed to have arisen. Following two-way interspecific hybridisation of filamentous fungi, segregation of mt genomes is believed to result in pure unmixed derivatives, although the temporary presence of heteroplasmons may permit intergenomic recombination events [24]. Mt DNA also offers an advantage in terms of copy number, which has been estimated to range from ten to several thousand per cell [25,26]. Consequently, depth-of-coverage related to mt DNA in a whole genome sequencing dataset will be considerably higher than for genomic regions, increasing confidence of analysis. Furthermore, comparisons of molecular evolution between the nuclear and mitochondrial genomes of fungi have revealed accelerated rates in the latter [27], potentially increasing the capacity to discriminate between closely related taxa.

The present study describes phylogenetic analysis of fescue-derived endophytes based on three nuclear protein-coding genes (*tefA*, *tub2*, *perA*) and the complete protein-coding gene complement of the mt genome. This study has provided confirmation of known relationships and additional novel insights, due to the higher resolution permitted by multiple gene analysis. The majority of previous studies of *Epichloë*-*Neotyphodium* species have been based on sequences from PCR amplicons of partial gene sequences. In contrast, the present study describes, for the first time, identification and use of complete sequences from the relevant genes solely derived from whole genome sequence datasets.

## Methods

### Endophyte isolates and DNA extraction

Phylogenetic analysis was performed on 16 endophyte isolates (Table 1) representing the known taxa *N. coenophialum*, *N. uncinatum*, *Fa*TG-2 and *Fa*TG-3, as well as the two putative distinct taxa previously designated as *Fa*TG-3-like and UNS [9]. Genomic DNA was extracted from lyophilized mycelia by cetyltrimethylammonium bromide (CTAB) extraction [28], and the quality and quantity of the DNA was assessed by both agarose gel electrophoresis and specific absorbance measurements using the NanoDrop 2000 Spectrophotometer (Thermo Scientific, Waltham, Massachusetts, USA).

**Table 1:** Endophyte isolates used for phylogenetic analysis

**Endophyte isolate**	**Species or taxon**	**Host species**	**Origin**	Source*
E34	*N. coenophialum*	*F. arundinacea*		RBG
NEA14	*N. coenophialum*	*F. arundinacea*	France	NZA
NEA16	*N. coenophialum*	*F. arundinacea*	France	NZA
NEA20	*N. coenophialum*	*F. arundinacea*	France	NZA
NEA22	*N. coenophialum*	*F. arundinacea*	Spain	NZA
NEA21	*Fa*TG-3^#^	*F. arundinacea*	Morocco	NZA
NEA23	*Fa*TG-3	*F. arundinacea*	Tunisia	NZA
NEA17	*Fa*TG-2^$^	*F. arundinacea*	Spain	NZA
NEA32	*Fa*TG-2	*F. arundinacea*	Morocco	NZA
NEA19	UNS	*F. arundinacea*	Algeria	NZA
NEA18	UNS	*F. arundinacea*	Sardinia	NZA
E81	*N. uncinatum*	*F. pratensis*		RBG
NEA33	*Fa*TG-3-like	*F. arundinacea*	Morocco	NZA
9707	*E. baconii*	*Agrostis tenuis*	Switzerland	ETH Zürich
9340	*E. typhina*	*Poa pratenis*	Switzerland	ETH Zürich
9636	*E. typhina*	*Poa trivialis*	Switzerland	ETH Zürich
SE	*N. lolii*	*Lolium perenne*	New Zealand	DEPI

*ETH Zürich: Eidgenössische Technische Hochschule Zürich (Swiss Federal Institute of Technology, Zürich, Switzerland); NZA: New Zealand Agriseeds Ltd, Christchurch, New Zealand; RBG: Royal Barenbrug Group, Nijmegen-Noord, Netherlands; DEPI: Department of Environment and Primary Industries, Victoria, Australia.

^$^*Fa*TG-2: *Festuca arundinacea* taxonomic group 2.

^#^*Fa*TG-3: *Festuca arundinacea* taxonomic group 3.

### Paired-end library preparation and sequencing

Genomic DNA was fragmented in a Covaris instrument (Woburn, MA, USA) to an average size of 100–900 bp. For each endophyte DNA sample, paired-end libraries with inserts c. 400 bp in size were prepared using the standard protocol (TruSeq DNA Sample Prep V2 Low Throughput: Illumina Inc., San Diego, USA) with paired-end adaptors. Library quantification was performed using the KAPA library quantification kit (KAPA Biosystems, Boston, USA). Paired-end libraries were pooled according to the attached adaptors and sequence analysed using the HiSeq2000 platform (Illumina) following the standard manufacturer’s protocol.

### Processing and assembly of sequence data

All generated sequence reads were quality controlled by filtering and trimming of reads based on quality using a custom Python script, which calculates quality statistics, and stores trimmed reads in several fastq files. Data assembly was performed using the Linux-based *de novo* assembler Velvet ver.1.1.06 [29]. For Velvet assembly, different hash lengths (K-mer sizes) ranging from 39 to 51 were tested as appropriate for different sequence read sets, and the minimum contig length was always defined as 200 bp. Values for estimated coverage and coverage cut-off were set to auto.

### Assembly of nuclear gene sequences

Presence and copy number of *tub*2, *tef*A, and *per*A genes (using Genbank accession numbers:*tub2*: AY722412, *tefA*: FJ660614, *perA*: AB205145) in each endophyte genome were initially determined through nucleotide BLAST (Basic Local Alignment Search Tool) [30] analysis using contigs from the optimised Velvet assembly of total reads as the database. In order to assemble each gene copy, matching reads for each reference gene were identified from a database of all trimmed reads from each endophyte genome, through a similarity search using BLAST, defining the E value threshold as 0.1. From this BLAST output, all corresponding paired reads (both forward and reverse) were extracted from the database, and the second reads were reverse-complemented. Each of the first and second reads was concatenated and used as BLASTN queries against a database consisting solely of each reference gene sequence for assembly. From this search, reads that matched in an anti-sense orientation were reverse-complemented in order to orientate the concatenated reads in 5′- to 3′-orientation against the reference gene sequence. Subsequently, reads were separated into two distinct sets (designated read1 and read2) and the two groups were used individually as BLASTN queries against the gene sequence that was to be assembled. The aim of these individual BLAST searches was to estimate positions for each read against the reference database sequence. After assigning positions, each read was padded to the appropriate position along the reference gene sequence using a customised PERL script. The padded read pairs (when both reads were selected from the BLAST output) were then concatenated and saved in FASTA format. Using the graphical multiple sequence editor SeaView [31], the padded reads were manually assembled into the defining gene sequences. Functional sequences were predicted for multiple *per*A gene copies through translation of each gene sequence using ExPASy online DNA sequence translation tool [32].

### Phylogenetic analysis of nuclear genes

Multiple alignments of the complete gene sequences for each selected gene were performed individually using ClustalW [33] with default parameters. To reconstruct tree topology, parsimony, maximum likelihood (ML) and neighbour-joining (NJ) methods were used as implemented in MEGA 5 [34] with default parameters and 1,000 bootstrap replicates. Gene sequences available in Genbank from related endophyte species were used appropriately, and corresponding accession numbers are provided in each tree diagram. After identification of proposed origin from an individual genome for each gene copy based on the individual gene trees, the three nuclear genes were concatenated in the order *tub*2-*tef*A-*per*A and aligned using ClustalW with the default parameters. For the concatenated multiple sequence alignment, phylogenetic topology was reconstructed using MEGA with default parameters and 1,000 bootstrap replicates. Phylogenetic networks were constructed for aligned concatenated gene sequences using the NeighborNet algorithm [35] on the Nei–Li pairwise distance matrix, and network diagrams were produced using the program SplitsTree4 [36].

### Assembly of mitochondrial genomes

Contigs of mitochondrial (mt) genome origin were initially identified using nucleotide BLAST at an E value threshold of 0.001 through alignment of a database containing all contigs from the optimised Velvet assembly (as described above) against the mt genome of the *N. lolii* standard endophyte (SE) strain as a reference [37] (Genbank accession number KF906135). For each candidate endophyte, a set of contigs with higher read depth coverage were shown to have a significant match to the reference mt genome sequence. A cut-off value for read depth coverage of each mt genome was identified based on the BLAST output, and a second Velvet assembly was performed with assignment of this value as the coverage cut-off value in order to filter contigs derived from the mt genome. A range of k-mer values were tested, and a final assembly was accepted on the basis of features such as total number of assembled contigs, N50 value and cumulative contig length. For those mt genomes with few (2–5) contigs, ordering was performed with BLASTN (E value threshold of 0.001) based on the pre-existing SE mt genome sequence, and overlapping regions were manually linked. In order to confirm gaps observed in comparison to the SE mt genome, alignment was performed for trimmed reads using the Burrows-Wheeler Alignment (BWA) tool [38] with the maximum number of gap openings set to five. Mapped reads were viewed using Tablet 1.12.02.06 [39], a graphical viewer for sequence alignment. Observed gaps were further confirmed through grouping of observed gap positions within each endophyte species or taxon.

Identification of protein-coding gene sequences was performed using each mt genome sequence as the query database against the individual mitochondrial protein gene sequences from the clavicipitacean entomopathogenic fungus *Metarhizium anisopliae* (Genbank accession number NC008068). Identified protein-coding genes were concatenated according to the order observed in the *M. anisopliae* mt genome.

### Phylogenetic analysis of mt genome protein coding gene complement

Multiple alignment of concatenated mitochondrial protein-coding gene complements from the 19 endophytes and counterparts in *M. anisopliae* was performed using the M-LAGAN program within the mVISTA on-line suite of computational tools [40], with default parameters. Alignments were manually edited for mis-alignments that may have accumulated due to overlapping gene fragments. To reconstruct the tree topology, parsimony, ML and NJ methods were used as implemented in MEGA 5 with default parameters and 1,000 bootstrap replicates. Furthermore, to study the level of identity of each mt genome protein-coding complement relative to that of *M. anisopliae*, aligned sequences were visualized through use of a VISTA plot [41].

## Results

### Identification of individual nuclear gene copies

**Figure 1:**
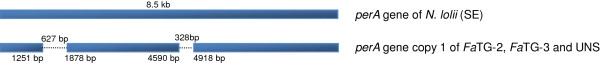
**Schematic diagram of *perA *** **gene copy 1 of *Fa*** **TG-2, *Fa*** **TG-3 and UNS aligned with the *perA *** **gene sequence of *N. lolii.***

Presence of the three nuclear genes (*tub2*, *tefA* and *perA*) was determined for 13 fescue-derived endophyte genomes and all other reference taxa (*Epichloë* spp. and *N. lolii*) that were used for this study. The observed number of copies for each gene within each taxon was then determined (Table 2). For each gene, 3 copies were observed within individual *N. coenophialum* genomes, while 2 copies were observed from all other taxa apart from *N. uncinatum*, which contained a single copy of the *tub2* gene. Moreover, one of the assembled *perA* gene copies from *Fa*TG-2, *Fa*TG-3 and UNS genomes revealed a common variant structure based on large- to moderate-sized deletions (coordinates 1251-1878 bp and 4590-4918 bp; Figure 1).

**Table 2:** Number of gene copies identified from each endophyte taxon represented in the analysis

	**Gene**	**Endophyte taxon**					
**Observed number of gene copies for**		*N. coenophialum*	*Fa*TG-2	*Fa*TG-3	*Fa*TG-3 like	UNS	*N. uncinatum*
	***perA***	3	2	2	2	2	2
	***tub2***	3	2	2	2	2	1
	***tefA***	3	2	2	2	2	2

### Phylogenetic relationships based on nuclear genes

**Figure 2:**
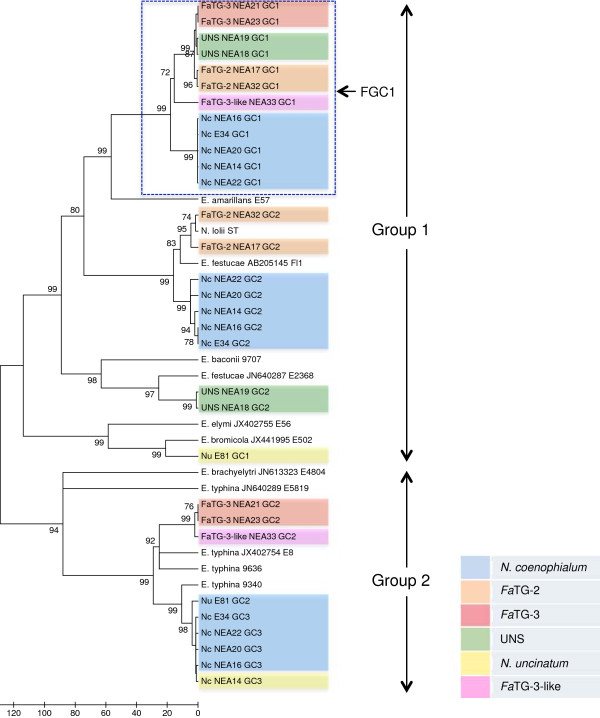
**Bootstrap consensus tree generated through parsimony analysis of *perA *gene sequence among reference endophyte isolates and selected fescue-derived endophytes.** Branches with bootstrap values of greater than 70% from 1000 bootstrap replication are marked next to each branch. Endophyte taxa are colour coded as indicated in the legend. Endophyte taxon abbreviations prior to isolate name are as follows: Nc = *N. coenophialum*, Nu = *N. uncinatum*, UNS = uncharacterised *Neotyphodium* species. The *perA* gene sequence of *E. amarillans* was derived from whole genome shotgun sequence available in GenBank under accession number: AFRF00000000. The GenBank accession numbers of the *perA* genes derived from fescue-derived endophytes are provided in Additional file 6.

**Figure 3:**
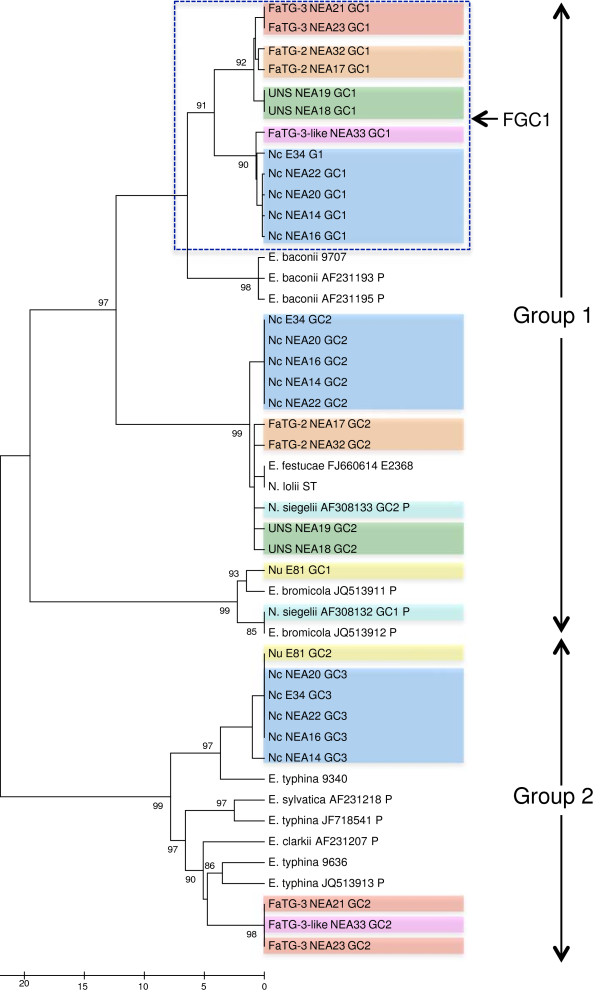
**Bootstrap consensus tree generated through parsimony analysis of *tefA *gene sequence among reference endophyte isolates and selected fescue-derived endophytes.** Diagram properties are as described for Figure 2. P indicates partial gene sequences obtained from the Genbank. Accessions numbers are provided adjacent to species name. The GenBank accession numbers of the *tefA* genes derived from fescue-derived endophytes are provided in Additional file 6.

**Figure 4:**
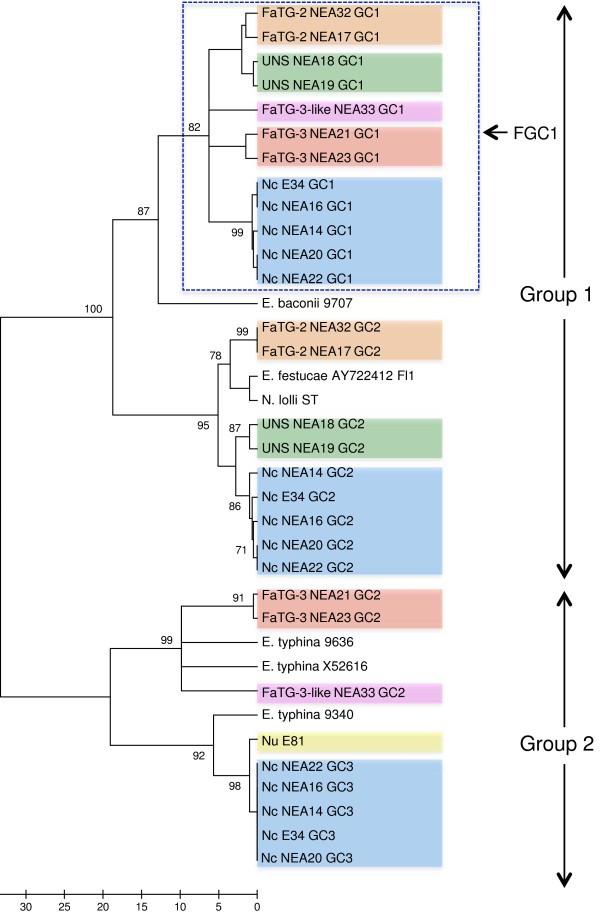
**Bootstrap consensus tree generated through parsimony analysis of *tub2 *gene sequence among reference endophyte isolates and selected fescue-derived endophytes.** Diagram properties are as described for Figure 2. The GenBank accession numbers of the *tub2* genes derived from fescue-derived endophytes are provided in Additional file 6.

Phylogenetic relationships were reconstructed based on individual gene sequences of *perA*, *tub2*, and *tefA* genes, as well as the concatenated sequences of all three genes, using parsimony ML and NJ methods (related alignments can be found at URL: http://purl.org/phylo/treebase/phylows/study/TB2:S14923). All positions containing gaps and missing data were eliminated during each analysis. Corresponding gene-specific DNA sequences from other taxa that were previously deposited in GenBank were included. Similar tree topologies were observed for all three methods, and the most parsimonious phylograms were selected (Figures 2, 3 and 4 and Additional files 1, 2, 3, 4 and 5). The GenBank accession numbers of the nuclear genes derived from fescue-derived endophytes are provided in Additional file 6. Maximum parsimony analysis based on the individual gene sequences of the three genes resolved more than one most parsimonious tree, and so bootstrap consensus trees inferred from 1000 replicates are displayed for each gene. In each phylogram, individuals from the same endophyte taxa were clustered together, and were separated from the *Epichloë* isolates that were used in the study. Apart from the placement of *E. baconii* and *E. amarillans* within the *perA*-and *tefA*-specific trees, phylogenetic analysis of individual genes resolved similar genomic relationships between endophyte taxa. All major clades that were defined by single gene phylogeny were also strongly supported by the phylogeny of the concatenated *perA*, *tub2*, and *tefA* genes, due to observation of similar tree topology. Maximum parsimony analysis of concatenated genes yielded a single most parsimonious tree with high level of bootstrap support for the majority of the individual branches.

In all instances, the phylogenetic trees were predominantly separated into two major groups representing different *Epichloë* species. For example, in the *tefA* gene-specific phylogram (Figure 3), Group 1 contained the taxa *E. festucae*, *E. baconii*, *E. amarillans* and *E. bromicola*, while Group 2 contained *E. sylvatica*, *E. typhina*, and *E. clarkii*. Furthermore, individual gene copies from the fescue-derived endophytes were located closely adjacent to the reference *E. festucae-*, *E. bromicola-* and *E typhina-*derived sequences, suggesting affinities to putative sub-genome components of the heteroploid taxa. However, in all instances members of the fescue-derived endophyte (*N. coenophialum*, *Fa*TG-2, *Fa*TG-3, *Fa*TG-3-like and UNS) gene copy 1 (FGC1) clade were not so closely related to sequences from any of the currently included *Epichloë* endophytes (Figures 2, 3 and 4 and Additional file 2). In the *perA*-based phylogeny, *E. amarillans* formed a sister group to FGC1, while addition of a partial gene sequence from *E. amarillans* to the *tefA*-based phylogeny generated a separate clade also containing *E. baconii*. Nevertheless, in the absence of *E. amarillans,* the *tub2*-and concatenated gene-based analysis placed FGC1 as a sister group to *E. baconii*.

The genomes of the known heteroploid endophyte taxa *N. coenophialum*, *N. uncinatum* and *Fa*TG-3 contributed gene copies to both major phylogram groups, consistent with hybrid origins either from one of the relevant *Epichloë* species, or a closely related taxon. In contrast, the multiple gene copies from *Fa*TG-2 and UNS were located only within Group 1. High levels of similarity between sequences from different endophyte taxa suggested the presence of common sub-genomic components. For instance, *N. coenophialum* and *N. uncinatum* genotypes always contributed gene copies that were identical or very closely related to one another, and similar to those from *E. typhina*. Although *N. coenophialum* gene copies showed close affinities to those from other fescue endophyte taxa within Group 1, distinct clusters were generated in all instances. A further point of interest was the close relationships between the *Fa*TG-2-derived *perA* and *tefA* gene copies and those obtained from both *E. festucae* and its asexual anamorph, *N. lolii*.

**Figure 5:**
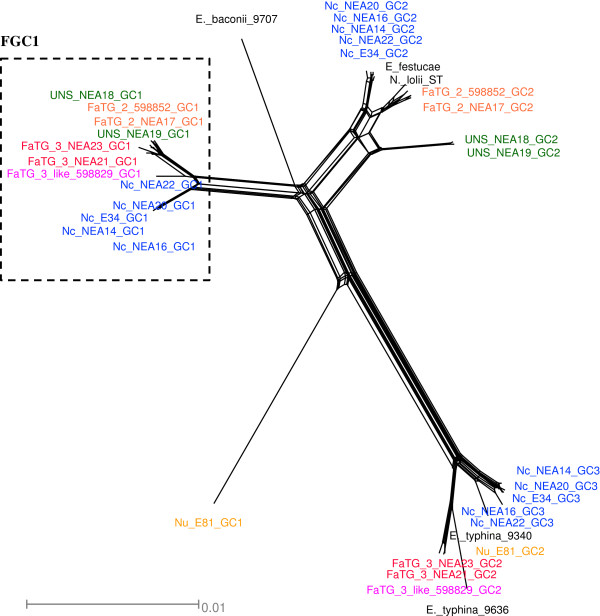
**NeighborNet network of relationships between copies of the concatenated *tub2*, *tefA *** **and *perA *** **gene sequences from reference endophyte isolates and selected fescue endophytes.**

As an alternative way to explore the reticulated evolutionary relationships between sexual *Epichloë* progenitor species and heteroploid *Neotyphodium* endophytes, a phylogenetic network diagram was constructed based on the concatenated nuclear gene sequence (Figure 5). Distinct clusters for asexual endophyte-derived gene copies were formed around the reference *E. festucae* and *E. typhina* sequences, corresponding to respective clades in Group 1 and Group 2 of the phylograms, and further supporting the hypothesis of progenitor relationships. Separation of *E.baconii* from FGC1 gene copies was also consistent with the phylogram structure. The network analysis also served to further demonstrate the differentiation of *Fa*TG-2 and UNS-derived gene copies within the *E. festucae*-containing clade of the phylograms, despite similarity in both instances to *E. festucae*. Similar results were obtained for *Fa*TG-3- and *Fa*TG-3-like-derived gene copies corresponding to members of FGC1 within the phylograms, and, to a lesser extent, Group 2.

Putative functional gene copies for *perA* gene were predicted based on sequence translation (to produce an intact biosynthetic enzyme) and assigned to putative progenitor origins based on phylogenetic affinities with *Epichloë* species that are known to contain such sequences (Table 3). Putative gene functionality was consistent between the predicted sub-genomic components of each taxon. For example, both *E. festucae-* and *E. typhina-*like *perA* gene copies (from Group 1 and Group 2) were predicted to be functional for all *N. coenophialum* isolates used for this study. In contrast, the *perA* gene copy characteristic of FGC1 was predicted to be non-functional for the *N. coenophialum*, UNS, *Fa*TG-2, *Fa*TG-3, and *Fa*TG-3-like gene copies. Furthermore, predicted gene functionality was also consistent with the results of preliminary alkaloid profile analysis. For example, both *perA* gene copies of UNS endophytes were predicted to be non-functional, and these endophytes have not been observed to produce peramine *in planta* (P. Ekanayake, unpublished).

**Table 3:** **Summary of gene translation studies for *perA *** **gene**

**Endophyte taxon**	**Predicted functional gene copies**	**Results of gene translation relation to proposed progenitor origin**
*N. coenophialum*	2 (3)	E. f (F), E. t (F), FGC1 (NF)
*Fa*TG-2	1 (2)	E. f (F), FGC1 (NF)
*Fa*TG-3	1 (2)	E. t (F), FGC1 (NF)
*Fa*TG-3-like	1 (2)	E. t (F), FGC1 (NF)
UNS	0 (2)	E. f (NF), FGC1 (NF)
*N. uncinatum*	1 (2)	E. t (F), E.br (NF)

Note: Total number of gene copies identified for each taxon is given in brackets next to the number of proposed functional gene copies. E.f = *E. festucae*, E. t = *E. typhina*, FGC1 = Fescue gene copy 1, E. br = *E. bromicola*, F = predicted functional gene, NF = predicted non-functional gene.

### Mitochondrial genome sequence structure

General structural characteristics for the 19 mt genomes were determined (Table 4), revealing variation of overall size from 51,884 - 96,481 bp. All except *E. typhina* mt genome sizes varied slightly within a given taxon, larger differences being observed between taxa. All shared the same 13 protein-coding genes arranged in the same order, accounting for 15%-28% of the entire mt genome, and showing 90% cumulative sequence similarity to the out-group species, *M. anisopliae*. In contrast to conservation of the protein-coding components, higher levels of sequence divergence were apparent within the intergenic regions, due to multiple insertion/deletion events when compared to the *N. lolii* SE mt genome that was used as a reference. A further complication in this analysis was the presence of nuclear genome-derived sequences that showed more distant affinities to the mt DNA, perhaps generated by inter-organelle transfer and integration.

**Table 4:** Comparison of mt genomes of analysed endophytes

**Sample name**	**Endopphyte taxon**	**Number of contigs assembled**	**Coverage**	**Mt Genome**		
				**Total length (bp)**	**% protein gene content**	**% ID within protein gene content**
NC008068	*M. anisopliae*	N/A	N/A	24673	59.11	-
NEA22	*N. coenophialum*	4	>800	93,968	15.52	90.7
NEA14	*N. coenophialum*	5	>900	94,628	15.41	90.5
NEA16	*N. coenophialum*	1	>1800	95,628	15.25	90.9
NEA20	*N. coenophialum*	5	>1700	94,924	15.36	90.7
E34	*N. coenophialum*	4	>2100	95,719	15.24	90.9
NEA18	UNS	4	>1200	82,857	17.60	91.0
NEA19	UNS	1	>2400	83,619	17.44	90.7
NEA23	*Fa*TG-3	4	>2800	91,949	15.86	90.7
NEA21	*Fa*TG-3	1	>700	91,820	15.88	90.8
598829	*Fa*TG-3 like	1	>2500	79,875	18.26	90.8
NEA17	*Fa*TG-2	2	>1200	95,822	15.22	91.2
598852	*Fa*TG-2	4	>3800	96,481	15.12	91.2
E81	*N. uncinatum*	3	>970	84,718	17.22	90.6
9636	*E. typhina*	1	>2600	85,030	17.15	90.6
9340	*E. typhina*	2	>2500	51,884	28.11	90.5
9707	*E. baconii*	1	>1400	61,133	23.86	89.9
	*E. festucae*	N/A	N/A	69696	20.93	91.1
SE	*N. lolii*	N/A	N/A	88738	16.44	91.0
NEA11	*Lp*TG-2	N/A	N/A	88810	16.42	91.1

### Phylogenetic relationships based on mitochondrial genome comparisons

**Figure 6:**
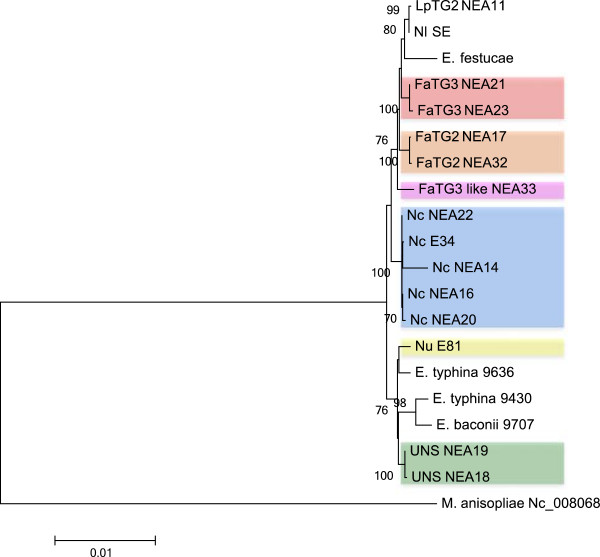
**Bootstrap consensus tree generated through maximum likelihood analysis of concatenated protein-coding gene sequences within the mitochondrial genomes of reference endophyte isolates and selected fescue endophytes.** Diagram properties are as described for Figure 2. The tree was rooted by addition of the mt genome protein-coding gene complement of *Metarhizium anisopliae*.

Phylogenetic relationships were reconstructed based on the concatenated sequences of 13 mitochondrial protein-coding genes from the fescue endophytes, while the equivalent for *M. anisopliae* was used as the out-group (Figure 6). Similar tree topology was observed for parsimony, ML and NJ methods (related alignments can be found at URL: http://purl.org/phylo/treebase/phylows/study/TB2:S14923). As expected, individual sequences from the same taxon clustered together. A number of putative progenitor relationships, such as that between *E. typhina* and *N uncinatum,* were more readily apparent from the phylogram. Close relationships were revealed between the *N. lolii* and *Lp*TG-2 mt genomes and that of their putative sexual progenitor, *E. festucae*, and similar, but less close, relationships were apparent for *N. coenophialum* and *Fa*TG-2. Commonalities of mitochondrial genome structure were evident between the *Fa*TG-3 and *Fa*TG-3-like mt genomes, albeit with lower bootstrap support, but affinity to potential *Epichloë* genomes was not so obvious, although an *E. festucae* mt genome provides the most obvious candidate. This result was inconsistent with the data from the nuclear gene analyses in the present study, that revealed closer relationships of gene copies from the *Fa*TG-3 and *Fa*TG-3-like isolates to the putative FGC1 progenitor, and to *E. typhina*. A clear differentiation of the UNS mt genome from that of the preceding groups, with higher levels of sequence similarity to *E. baconii* and *E. typhina* rather than *E. festucae,* was evident from this analysis*.*

## Discussion

The results obtained in the present study have confirmed the accuracy of previous assignment of endophyte accessions to distinct known taxonomic groups based on SSR polymorphism, along with the definition of several putative novel taxa [9]. The prior study was only capable of performing phenetic classification, but analysis of individual nuclear gene sequences has further permitted exploration of genome complexity within the heteroploid endophyte taxa, as well as interpretation of relationships with contemporary *Epichloë* species as representatives of putative progenitors.

Following the assembly of Illumina HiSeq2000 short reads utilising the Velvet assembly algorithm it was observed that large number of contigs were generated for heteroploid fescue grass-derived endophyte genomes (see assembly statistics listed in Additional file 7). This observation indicated that although Velvet is well-suited to assembly of haploid genomes, is not so appropriate for heteroploid genomes. Furthermore, in those instances characterised by multiple gene copies, Velvet was incapable of constructing the individual gene copies using short reads. However, the number of assembled contigs was sufficient to indicate the number of gene copies, and when evidence for multiple copies was obtained, individual genes were accurately assembled by a manual process.

### Copy number variation of selected nuclear genes

The presence of 3 copies for each of the *tefA*, *tub2* and *perA* genes in the *N. coenophialum* genome suggests a tri-parental hybrid origin, consistent with previous studies [17,23]. Similarly, the observation of 2 copies for each gene in the genomes of other heteroploid endophyte taxa (*Fa*TG-2, *Fa*TG-3, *Fa*TG-3-like and UNS) is compatible with a series of bi-parental hybrid origins. Although *N. uncinatum* has also previously been inferred to have arisen as a bi-parental hybrid, the presence of a sole *tub2* gene copy suggests selective gene loss, as previously proposed to account for the current heteroploid constitution of this and other taxa. These results, apart from concordance with earlier sequence-based studies [14,19], are also consistent with the complexity of SSR profiles from the same accessions [9], which typically contained up to 3 distinct amplicons from *N. coenophialum* genotypes, and up to 2 amplicons from the other taxa in this study.

### Phylogeny of previously described fescue-derived endophytes

**Figure 7:**
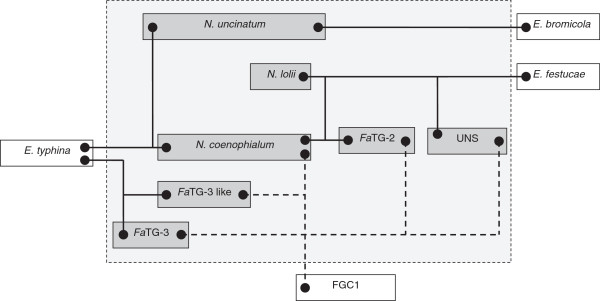
**Summary of nuclear genome affinities between endophyte taxa.** In the graphic, proposed progenitors are represented as species outside the box. Solid lines indicate the clear relationships with characterised progenitor species, while lower confidence relationships to uncharacterised progenitors are marked by dashed lines.

The present study has also permitted identification of those *Epichloë* species that are likely to be most closely related to the taxa that participated in hybrid origins. Previous phylogenetic studies based on two of the nuclear genes used in this study (*tub2* and *tefA*), as well as the *act*1 actin gene*,* have provided evidence for progenitor identity [15]. Three tall fescue-derived endophyte taxa have previously been included in such studies, *N. coenophialum* was proposed to have originated from *E. festucae*-, *E. baconii*- and *E. typhina*-like ancestors [18,19,42], while *Fa*TG-2 and *Fa*TG-3 were suggested to be derived from *E. festucae-* and *E. baconii-*like, and *E. baconii-* and *E. typhina*-like ancestors, respectively [17]. As summarised in Figure 7, the present study was consistent with these predictions in terms of affinities to contemporary *E. festucae* and *E. typhina* genotypes, but more distant relationships were observed for *E. baconii*. The group designated FGC1 in this study, which cannot be unequivocally attributed to a *E. baconii*-like progenitor, was also identified in a previous study and termed the ‘*Lolium*-associated endophyte’ (LAE) clade [23,43]. Furthermore, two distinct *E. typhina* lineages appear to have contributed to formation of the *N. coenophialum/N. uncinatum* and *Fa*TG-3/*Fa*TG-3-like heteroploid genomes, respectively (Figure 7), based on interpretation of the tree and network diagrams.

Phylogenetic reconstruction based on the *perA* gene sequence revealed a closer relationship between *E. amarillans* and the FGC1 than for the *E. baconii* genotype that was used in this study. However, the *E. amarillans*-derived *tefA* gene sequence demonstrated a close genetic relationship to *E. baconii*, and *E. amarillans* formed a sub-clade with *E. baconii*, *E. festucae*, and *N. lolii* as well the FGC1 clade, consistent with previous studies [43]. Observed anomalies between the gene-specific phylogenies in the present study may be due to different rates of molecular evolution between endophyte-specific (*perA*) and housekeeping (*tefA* and *tub2*) genes. Further to this, addition of the entire *tefA* gene sequences of *E. amarillans* to the phylogenetic analysis may provide a higher level of resolution.

### Phylogeny of mt genomes

For *N. coenophialum*, *Fa*TG-2 and *Fa*TG-3, the mitochondrial gene complement-based analysis revealed closest relationships to *E. festucae*, suggesting that this or a closely related sexual species donated the cytoplasmic genome to the known heteroploid taxa. This conclusion is again consistent with previous studies, apart from the status of *Fa*TG-3, which does not show strong similarity to the mt genomes of either *E. baconii* or *E. typhina*. This anomaly may be due to the effects of recombination between progenitor mitochondrial genomes following generation of a heteroplasmon by parasexual processes [24]. Such mechanisms have been demonstrated to operate in sexual crosses between *E. typhina* endophytes, although in general, uniparental inheritance is observed [44]. Alternatively, accelerated evolutionary rates of mt DNA relative to nuclear DNA, which have been observed in animals, fungi and in certain protist species [27], may contribute to lower phylogenetic affinities. In support of this explanation, substantial size differences were observed between the mt genomes of the two *E. typhina* isolates used for this study, suggesting that extensive surveys of intraspecific diversity may be required to identify suitable candidates for progenitor status. However, mt genomes within a given heteroploid taxon were relatively uniform in size, suggesting that limited opportunities for evolutionary divergence have arisen.

In contrast, the *N. uncinatum* mt genome protein coding gene complement is very closely related to that of one *E. typhina* lineage, consistent with an origin from this species during heteroploid formation*.* Furthermore, the mt genome of *Lp*TG-2, which was inferred to have arisen as an *N. lolii* x *E. typhina* hybrid [45], demonstrates a high genetic similarity to the *N. lolii* mt genome, as confirmed by previous studies [37,46]. This latter observation suggests a relatively recent origin in evolutionary time, and the absence of complicating factors such as recombination between mt genomes.

### Phylogeny of novel fescue-derived endophytes

A close relationship was apparent between the previously described taxon *Fa*TG-2 and the novel UNS endophyte group, both of which show affinities to *E. festucae* and to the putative progenitor of the FGC1/LAE lineages. This result was consistent with the SSR-based phenetic analysis, in which *Fa*TG-2 and UNS accessions were located in sister groups within the same super-cluster of the phenogram [9]. Despite this close affinity, the present study was able to confirm that these two taxa are distinct, based on the formation of separate sub-clusters in both the FGC1 and Group 1 *E. festucae*-containing clades of the phylograms, as well as in the network diagram. The mt genome phylogram reinforced this distinction, suggesting that the UNS mt genome may have been contributed by the ancestor of the FGC1/LAE lineages, while the *Fa*TG-2 mt genome, as previously described, is most closely related to *E. festucae*. In combination, the data suggests that these two heteroploid taxa may be derived from hybridisation events in reciprocal mode between two pairs of closely related haploid species, or that divergence from common origins has occurred within each lineage.

Similarly, close relationships between the *Fa*TG-3 and *Fa*TG-3-like endophytes was revealed through the initial endophyte-specific SSR analysis [9]. In the present study, both *Fa*TG-3 and *Fa*TG-3-like endophytes display phylogenetic affinities to both *E. typhina* and the FGC1/LAE ancestor, with particularly strong similarity for the *E. typhina*-like gene copies. Similar relationships have been obtained for *Fa*TG-3-like endophytes (later designated as *Fa*TG-4) in a previous study of *tub2* phylogeny [14]. Furthermore, both groups also showed *E. festucae*-like mt genome structure. However, the two groups were identified in differing host grass taxa, *Fa*TG-3 genotypes being detected in Mediterranean hexaploid tall fescue accessions, while *Fa*TG-3-like endophytes were obtained from decaploid tall fescue [9]. At the genomic level, commonly observed deletions of the FGC1/LAE *perA* gene copy in *Fa*TG-3 were not present in the *Fa*TG-3-like endophytes. In general, the FGC1 copies of each nuclear gene were distinct between *Fa*TG-3 and *Fa*TG-3-like endophytes, suggesting origin of this genomic sub-component from related but distinct taxa.

### Variation of nuclear gene structure

Previous phylogenetic studies of *tub2*, *tefA*, *act*1 and several alkaloid biosynthesis genes, including *perA* were performed by PCR amplification and subsequent sequencing of amplified PCR products [17,21,23]. More recently, a study of *tub2* phylogeny made partial use of whole genome sequence data [13]. In contrast, the present study was solely based on whole genome sequencing and subsequent independent assembly of entire *tub2*,*tefA*, and *perA* genes, allowing comprehensive identification of insertion-deletion events. Two common deletions were identified in the FGC1-specific *perA* gene copies from *Fa*TG-2, *Fa*TG-3 and UNS endophytes. A previous study of *perA* gene phylogeny reported the presence of a 328 bp deletion within the coding region of the *Fa*TG-2 genome [23], similar to observations of the *Fa*TG-2, *Fa*TG-3 and UNS genomes in this study, and an additional adjacent deletion of 627 bp within the same gene copy was here identified for all three taxa. As *Fa*TG-2 endophytes have previously been demonstrated to effectively produce the alkaloid, *perA* gene function must be due not to the FGC-1 gene copy, but the alternate copy that is putatively derived from an *E. festucae*-like progenitor.

In addition to these structural changes, the *perA* sequence of *E. baconii* endophyte 9707 exhibited an identical deletion to that reported in the *E. festucae* endophyte E2368 [47], through loss of the reductase domain-encoding sequence at the 3′-terminus. Although this deletion is common among endophytes [3] none of the sequenced novel fescue endophytes were found to contain this deletion.

## Conclusion

Complex and highly reticulated evolutionary relationships between *Epichloë-Neotyphodium* endophytes have been predicted on the basis of multiple nuclear genes and entire mitochondrial protein-coding gene sequences derived from independent assembly of whole genome sequence reads. Furthermore, results from the present study have confirmed the distinct status of the novel fescue endophyte taxa *Fa*TG-3-like and UNS [9]. The designation of the *Fa*TG-3-like taxon as *Fa*TG-4, as proposed in a recent sequence-based phylogenomics analysis [14], is supported by the data presented here. For consistency, it is therefore also proposed that UNS is henceforth designated as *Fa*TG-5. Apart from fundamental implications for evolutionary processes, the present study has provided information and resources for detection, discrimination and potential modification of agronomically important endophyte taxa.

## Availability of supporting data

The data sets supporting the results of this article are included within the article and its additional files. Nuclear protein-coding sequences and the reference N. lolii mt DNA sequence have been deposited in GenBank. Sequence alignments of mitochondrial protein-coding genes have been deposited in TreeBASE at URL: http://purl.org/phylo/treebase/phylows/study/TB2:S14923.

## Competing interests

The authors declare that they have no competing interests.

## Authors’ contributions

PNE carried out the DNA extractions, generation of the DNA sequences, performed the phylogenetic analysis and drafted the manuscript. MR performed sequence analysis and assembly of the *N.lolii* SE mt genome. TIS, KMG, JWF and GCS co-conceptualised and coordinated the project, contributed to data interpretation and assisted in drafting the manuscript. All authors read and approved the final manuscript.

## Supplementary Material

Additional file 1 **Bootstrap consensus tree generated through parsimony analysis of *tub2 *gene sequence among extended set of reference endophyte isolates and selected fescue endophytes.** Branches with bootstrap values of greater than 70% from 1000 bootstrap replication are marked next to each branch. Endophyte taxa are colour coded as indicated in the legend. Endophyte taxon abbreviation prior to isolate name are as follows: Nc = *N. coenophialum*, Nu = *N. uncinatum*, UNS = uncharacterised *Neotyphodium* species. Click here for file

Additional file 2 **Phylogram obtained for parsimony analysis of concatenated gene sequences of *tub2, tefA *and *perA *among reference endophyte isolates and selected fescue endophytes.** Branches with bootstrap values of greater than 70% from 1000 bootstrap replication are marked next to each branch. Endophyte taxon abbreviations prior to isolate name are as follows: Nc = *N. coenophialum*, Nu = *N. uncinatum*, UNS = uncharacterised *Neotyphodium* species. Click here for file

Additional file 3 **Bootstrap consensus tree generated through maximum likelihood analysis of *tub2 *gene sequence among reference endophyte isolates and selected fescue endophytes.** Branches with bootstrap values of greater than 70% from 1000 bootstrap replication are marked next to each branch. Click here for file

Additional file 4 **Bootstrap consensus tree generated through maximum likelihood analysis of *tefA *gene sequence among reference endophyte isolates and selected fescue endophytes.** Branches with bootstrap values of greater than 70% from 1000 bootstrap replication are marked next to each branch. Click here for file

Additional file 5 **Bootstrap consensus tree generated through maximum likelihood analysis of *perA *gene sequence among reference endophyte isolates and selected fescue endophytes.** Branches with bootstrap values of greater than 70% from 1000 bootstrap replication are marked next to each branch. Click here for file

Additional file 6 Genbank accession numbers of the sequences represented in the phylogenetic study. Click here for file

Additional file 7 **Sequence output and *de novo *** **assembly statistics for sequenced fescue-derived endophytes.** Click here for file

## References

[B1] Arachevaleta M Bacon CW Hoveland CS Radcliffe DE Effect of the tall fescue endophyte on plant response to environmental stress Agron J 1989 13 83 90 10.2134/agronj1989.00021962008100010015x

[B2] Malinowski DP Belesky DP Adaptations of endophyte-infected cool-season grasses to environmental stresses: mechanisms of drought and mineral stress tolerance Crop Sci 2000 13 923 940 10.2135/cropsci2000.404923x

[B3] Schardl CL Young CA Faulkner JR Florea S Pan J Chemotypic diversity of epichloae, fungal symbionts of grassesfungal ecology 2012 13 331 344 10.1016/j.funeco.2011.04.005

[B4] Siegel MR Latch GCM Bush LP Fannin FF Rowan DD Tapper BA Bacon CW Johnson MC Fungal endophyte-infected grasses: alkaloid accumulation and aphid response J Chem Ecol 1990 13 12 3301 3315 10.1007/BF00982100 24263431

[B5] Schardl CL Grossman RB Nagabhyru P Faulkner JR Mallik UP Loline alkaloids: currencies of mutualism Phytochemistry 2007 13 980 996 10.1016/j.phytochem.2007.01.010 17346759

[B6] Tanaka A Tapper BA Popay A Parker EJ Scott B A symbiosis expressed non-ribosomal peptide synthetase from a mutualistic fungal endophyte of perennial ryegrass confers protection to the symbiotum from insect herbivory Mol Microbiol 2005 13 4 1036 1050 10.1111/j.1365-2958.2005.04747.x 16091042

[B7] Gallagher RT Hawkes AD Steyn PS Vleggaar R Tremorgenic neurotoxins from perennial ryegrass causing ryegrass staggers disorder of livestock: structure elucidation of lolitrem B J Chem Soc Chem Commun 1984 13 614 616

[B8] Porter JK Analysis of endophyte toxins - Fescue and other grasses toxic to livestock J Anim Sci 1995 13 3 871 880 7608022 10.2527/1995.733871x

[B9] Ekanayake PN Hand ML Spangenberg GC Forster JW Guthridge KM Genetic diversity and host specificity of fungal endophyte taxa in fescue pasture grasses Crop Sci 2012 13 2243 2252 10.2135/cropsci2011.12.0664

[B10] Hand ML Cogan NOI Forster JW Molecular characterisation and interpretation of genetic diversity within globally distributed germplasm collections of tall fescue (*Festuca arundinacea* Schreb.) and meadow fescue (*F. pratensis* Huds.) Theor Appl Genet 2012 13 1127 1137 10.1007/s00122-011-1774-6 22222441

[B11] Hand M Cogan N Stewart A Forster J Evolutionary history of tall fescue morphotypes inferred from molecular phylogenetics of the Lolium-Festuca species complex BMC Evol Biol 2010 13 1 303 10.1186/1471-2148-10-303 20937141 PMC2958922

[B12] Glenn AE Bacon CW Price R Hanlin RT Molecular phylogeny of *Acremonium* and its taxonomic implications Mycologia 1996 13 3 369 383 10.2307/3760878

[B13] Christensen MJ Leuchtmann A Rowan DD Tapper BA Taxonomy of *Acremonium* endophytes of tall fescue (*Festuca arundinacea*), meadow fescue (*F. pratensis*) and perennial ryegrass (*Lolium perenne*) Mycol Res 1993 13 9 1083 1092 10.1016/S0953-7562(09)80509-1

[B14] Schardl C Young C Pan J Florea S Takach J Panaccione D Farman M Webb J Jaromczyk J Charlton N Currencies of mutualisms: sources of alkaloid genes in vertically transmitted epichloae Toxins 2013 13 6 1064 1088 10.3390/toxins5061064 23744053 PMC3717770

[B15] Craven KD Blankenship JD Leuchtmann A Hignight K Schardl CL Hybrid fungal endophytes symbiotic with the grass *Lolium pratense* Sydowia 2001 13 1 44 73

[B16] Moon CD Scott B Schardl CL Christensen MJ The evolutionary origins of *Epichloë* endophytes from annual ryegrasses Mycologia 2000 13 6 1103 1118 10.2307/3761478

[B17] Moon CD Craven KD Leuchtmann A Clement SL Schardl CL Prevalence of interspecific hybrids amongst asexual fungal endophytes of grasses Mol Ecol 2004 13 1455 1467 10.1111/j.1365-294X.2004.02138.x 15140090

[B18] Tsai HF Liu JS Staben C Christensen MJ Latch GCM Siegel MR Schardl CL Evolutionary diversification of fungal endophytes of tall fescue grass by hybridization with *Epichloë* species Proc Natl Acad Sci USA 1994 13 7 2542 2546 10.1073/pnas.91.7.2542 8172623 PMC43405

[B19] van Zijll de Jong E Guthridge KM Spangenberg GC Forster JW Sequence analysis of SSR-flanking regions identifies genome affinities between pasture grass fungal endophyte taxa Int J Evol Biol 2011 Article ID 921312, 11 pages, doi:10.4061/2011/921312 10.4061/2011/921312 PMC3042632 21350638

[B20] Gentile A Rossi MS Cabral D KC D Schardl CL Origin, divergence, and phylogeny of *Epichloë* endophytes of native Argentine grasses Mol Phylogenet Evol 2005 13 196 208 10.1016/j.ympev.2005.01.008 15737591

[B21] Craven KD Hsiau PTW Leuchtmann A Hollin W Schardl CL Multigene phylogeny of *Epichloë* species, fungal symbionts of grasses Ann Mo Bot Gard 2001 13 1 14 34 10.2307/2666129

[B22] Schardl CL Young CA Hesse U Amyotte SG Andreeva K Calie PJ Fleetwood DJ Haws DC Moore N Oeser B Plant-symbiotic fungi as chemical engineers: multi-genome analysis of the clavicipitaceae reveals dynamics of alkaloid Loci PLoS Genet 2013 13 2 28 10.1371/journal.pgen.1003323 PMC3585121 23468653

[B23] Takach JE Mittal S Swoboda GA Bright SK Trammell MA Hopkins AA Young CA Genotypic and chemotypic diversity of *Neotyphodium* endophytes in tall fescue from Greece Appl Environ Microbiol 2012 13 5501 5510 10.1128/AEM.01084-12 22660705 PMC3406137

[B24] Griffiths AJF Mitochondrial inheritance in filamentous fungi J Genet 1996 13 403 414 10.1007/BF02966318

[B25] Gray MW Burger G Lang BF Mitochondrial evolution Science 1999 13 5407 1476 1481 10.1126/science.283.5407.1476 10066161

[B26] Gray MW Mitochondrial evolution Cold Spring Harb Perspect Biol 2012 13 9 10.1101/cshperspect.a011403 PMC3428767 22952398

[B27] Burger G Gray MW Franz Lang B Mitochondrial genomes: anything goes Trends Genet 2003 13 12 709 716 10.1016/j.tig.2003.10.012 14642752

[B28] Möller EM Bahnweg G Sandermann H Geiger HH A simple and efficient protocol for isolation of high molecular weight DNA from filamentous fungi, fruit bodies and infected plant tissue Nucleic Acids Res 1992 13 22 6115 6116 10.1093/nar/20.22.6115 1461751 PMC334490

[B29] Zerbino DR Birney E Velvet: algorithms for de novo short read assembly using de Bruijn graphs Genome Res 2008 13 5 821 829 10.1101/gr.074492.107 18349386 PMC2336801

[B30] Altschul S Gish W Miller W Myers E Lipman D Basic local alignment search tool J Mol Biol 1990 13 3 403 410 2231712 10.1016/S0022-2836(05)80360-2

[B31] Manolo G Stéphane G Olivier G SeaView Version 4: a multiplatform graphical user interface for sequence alignment and phylogenetic tree building Mol Biol Evol 2010 13 2 221 224 10.1093/molbev/msp259 19854763

[B32] Artimo P Jonnalagedda M Arnold K Baratin D Csardi G De Castro E Duvaud S Flegel V Fortier A Gasteiger E ExPASy: SIB bioinformatics resource portal Nucleic Acids Res 2012 13 W597 W603 10.1093/nar/gks400 22661580 PMC3394269

[B33] Larkin MA Blackshields G Brown NP Chenna R McGettigan PA McWilliam H Valentin F Wallace IM Wilm A Lopez R ClustalW and ClustalX version 2 Bioinformatics 2007 13 21 2947 2948 10.1093/bioinformatics/btm404 17846036

[B34] Tamura K Peterson D Peterson N Stecher G Nei M Kumar S MEGA5: molecular evolutionary genetics analysis using maximum likelihood, evolutionary distance, and maximum parsimony methods Mol Biol Evol 2011 13 2731 2739 10.1093/molbev/msr121 21546353 PMC3203626

[B35] Bryant D Moulton V NeighborNet: an agglomerative algorithm for the construction of phylogenetic networks Mol Biol Evol 2004 13 255 265 14660700 10.1093/molbev/msh018

[B36] Huson D Bryant D Application of phylogenetic networks in evolutionary studies Mol Biol Evol 2006 13 254 267 16221896 10.1093/molbev/msj030

[B37] Rabinovich M Genome structure and diversity in the perennial ryegrass (Lolium perenne l.) fungal endophyte Neotyphodium lolii 2011 Bundoora: La trobe University

[B38] Li H Durbin R Fast and accurate short read alignment with Burrows-Wheeler Transform Bioinformatics 2009 13 1754 1760 10.1093/bioinformatics/btp324 19451168 PMC2705234

[B39] Milne I Bayer M Cardle L Shaw P Stephen G Wright F Marshall D Tablet - next generation sequence assembly visualization Bioinformatics 2010 13 3 401 402 10.1093/bioinformatics/btp666 19965881 PMC2815658

[B40] Brudno M Do CB Cooper GM Kim MF Davydov E Green ED Sidow A Batzoglou S NISC Comparative Sequencing Program. LAGAN and Multi-LAGAN: efficient tools for large-scale multiple alignment of genomic DNA Genome Res 2003 13 4 721 731 10.1101/gr.926603 12654723 PMC430158

[B41] Frazer KA Pachter L Poliakov A Rubin EM I. D VISTA: computational tools for comparative genomics Nucleic Acids Res 2004 13 32 W273 279 15215394 10.1093/nar/gkh458 PMC441596

[B42] Moon CD Miles CO Jarlfors U Schardl CL The evolutionary origins of three new *Neotyphodium* endophyte species from grasses indigenous to the Southern Hemisphere Mycologia 2002 13 4 694 711 10.2307/3761720 21156542

[B43] Schardl CL Craven KD Speakman S Stromberg A Lindstrom A Yoshida R A novel test for host-symbiont codivergence indicates ancient origin of fungal endophytes in grasses Syst Biol 2008 13 3 483 498 10.1080/10635150802172184 18570040

[B44] Chung KR Leuchtmann A Schardl CL Inheritance of mitochondrial DNA and plasmids in the ascomycetous fungus, Epichloë typhina Genetics 1996 13 1 259 265 8770603 10.1093/genetics/142.1.259 PMC1206955

[B45] Kuldau GA Tsai HF Schardl CL Genome sizes of *Epichloë* species and anamorphic hybrids Mycologia 1999 13 5 776 782 10.2307/3761531

[B46] Schardl CL Leuchtmann A Tsai HF Collett MA Watt DM Scott DB Origin of a fungal symbiont of perennial ryegrass by interspecific hybridization of a mutualist with the ryegrass choke pathogen, Epichloë typhina Genetics 1994 13 4 1307 1317 8013907 10.1093/genetics/136.4.1307 PMC1205911

[B47] Fleetwood DJ Khan AK Johnson RD Young CA Mittal S Wrenn RE Hesse U Foster SJ Schardl CL B. S Abundant degenerate miniature inverted-repeat transposable elements in genomes of epichloid fungal endophytes of grasses Genome Biol Evol 2011 13 1253 1264 10.1093/gbe/evr098 21948396 PMC3227409

