# Supplementary figures and images for: Phylogenomics of fescue grass-derived fungal endophytes based on selected nuclear genes and the mitochondrial gene complement
Source: BMC Evol Biol. 2013 Dec 12;13:270. doi: 10.1186/1471-2148-13-270 (PMC4028799; doi:10.1186/1471-2148-13-270)

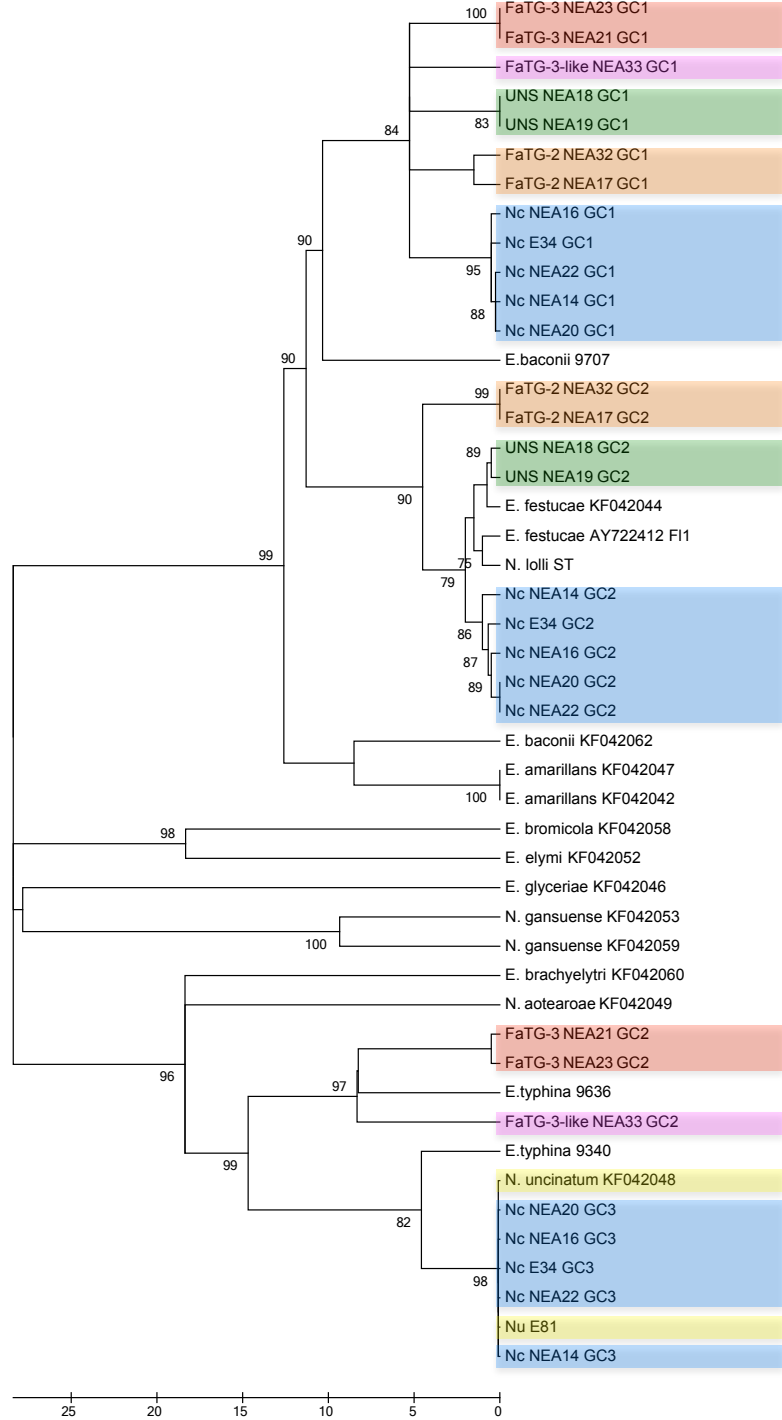

Supplement: Additional file 1 — Bootstrap consensus tree generated through parsimony analysis of tub2 gene sequence among extended set of reference endophyte isolates and selected fescue endophytes. Branches with bootstrap values of greater than 70% from 1000 bootstrap replication are marked next to each branch. Endophyte taxa are colour coded as indicated in the legend. Endophyte taxon abbreviation prior to isolate name are as follows: Nc = N. coenophialum, Nu = N. uncinatum, UNS = uncharacterised Neotyphodium species. [file 1471-2148-13-270-S1.pdf]

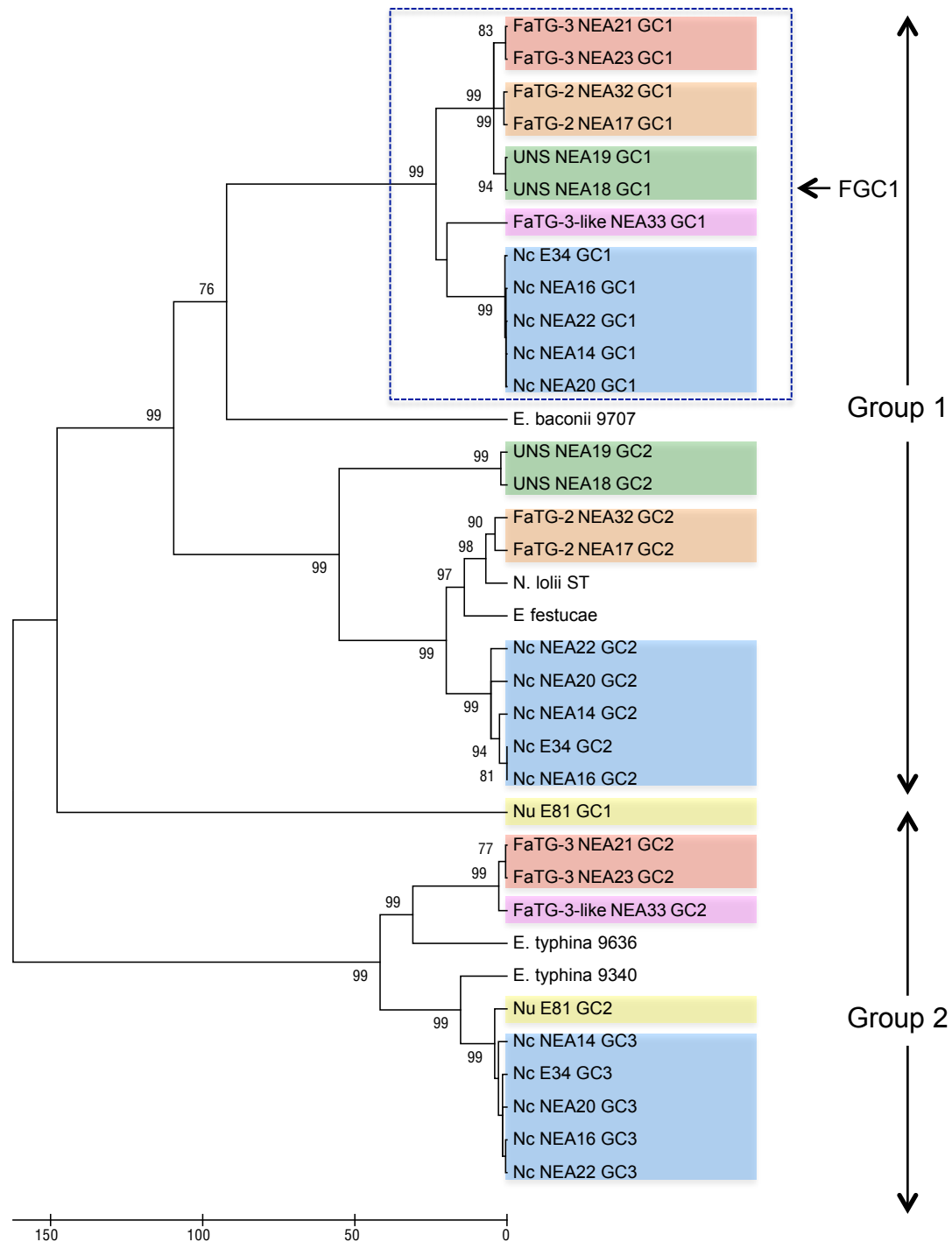

Supplement: Additional file 2 — Phylogram obtained for parsimony analysis of concatenated gene sequences of tub2, tefA and perA among reference endophyte isolates and selected fescue endophytes. Branches with bootstrap values of greater than 70% from 1000 bootstrap replication are marked next to each branch. Endophyte taxon abbreviations prior to isolate name are as follows: Nc = N. coenophialum, Nu = N. uncinatum, UNS = uncharacterised Neotyphodium species. [file 1471-2148-13-270-S2.pdf]

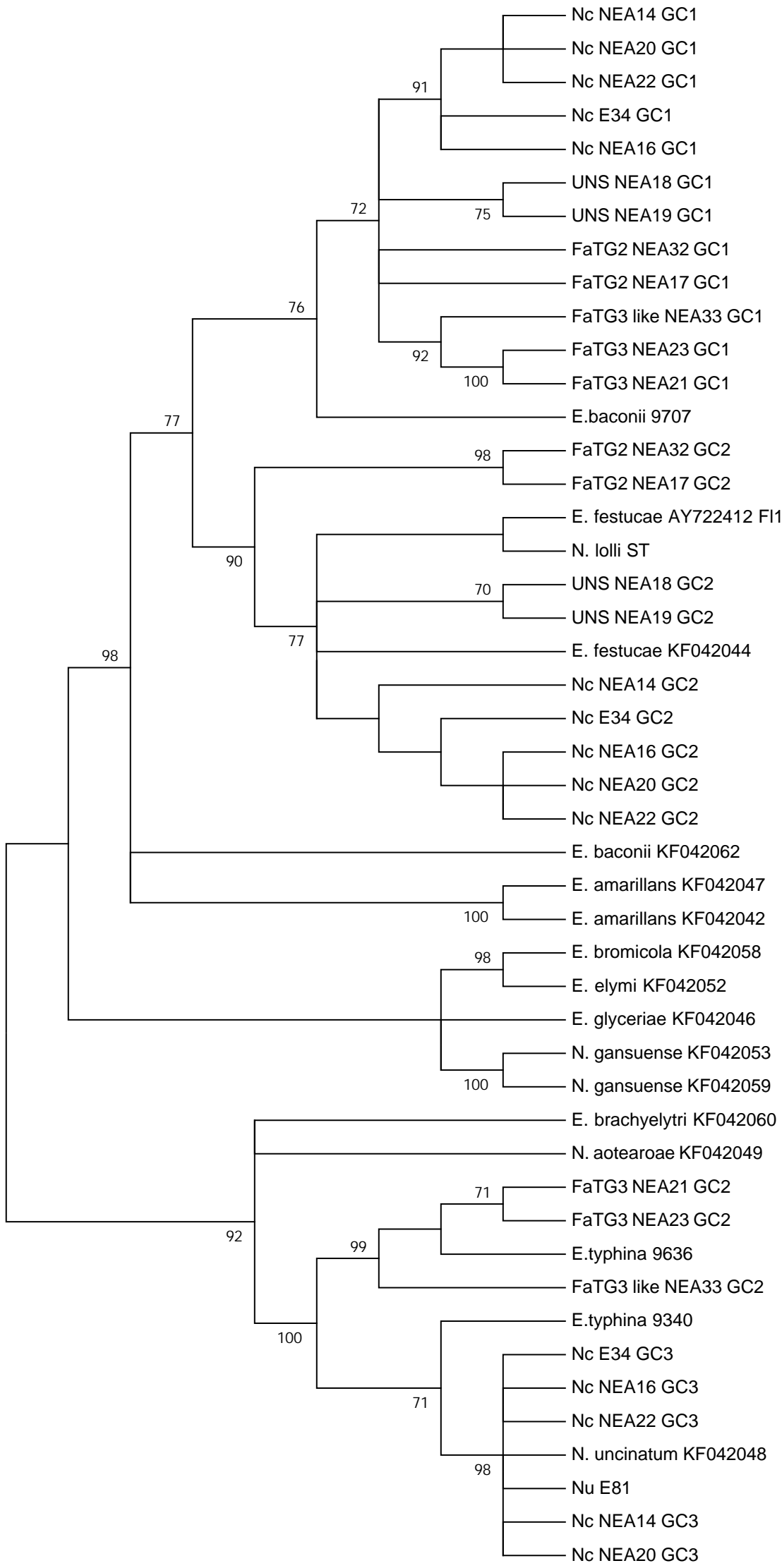

Supplement: Additional file 3 — Bootstrap consensus tree generated through maximum likelihood analysis of tub2 gene sequence among reference endophyte isolates and selected fescue endophytes. Branches with bootstrap values of greater than 70% from 1000 bootstrap replication are marked next to each branch. [file 1471-2148-13-270-S3.pdf]

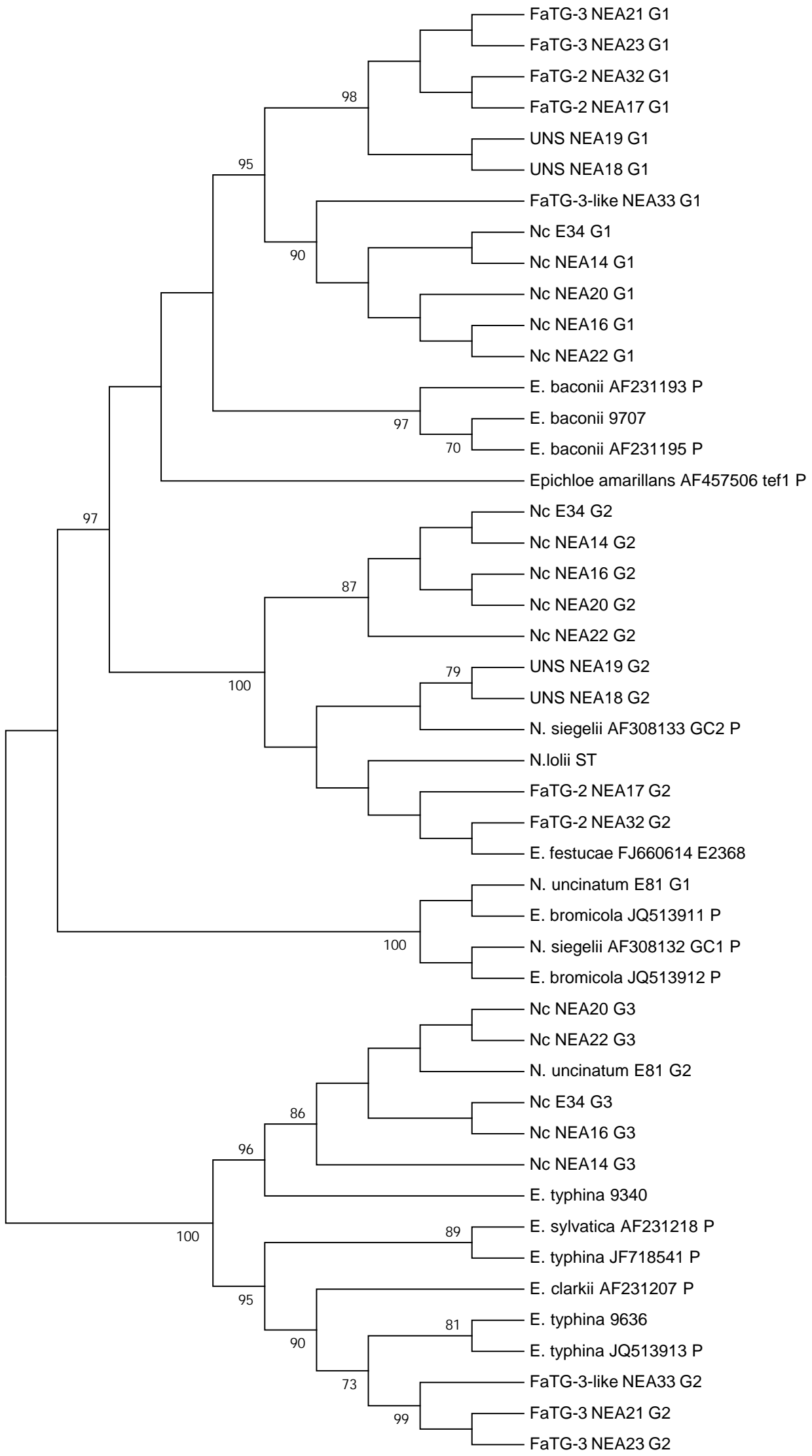

Supplement: Additional file 4 — Bootstrap consensus tree generated through maximum likelihood analysis of tefA gene sequence among reference endophyte isolates and selected fescue endophytes. Branches with bootstrap values of greater than 70% from 1000 bootstrap replication are marked next to each branch. [file 1471-2148-13-270-S4.pdf]

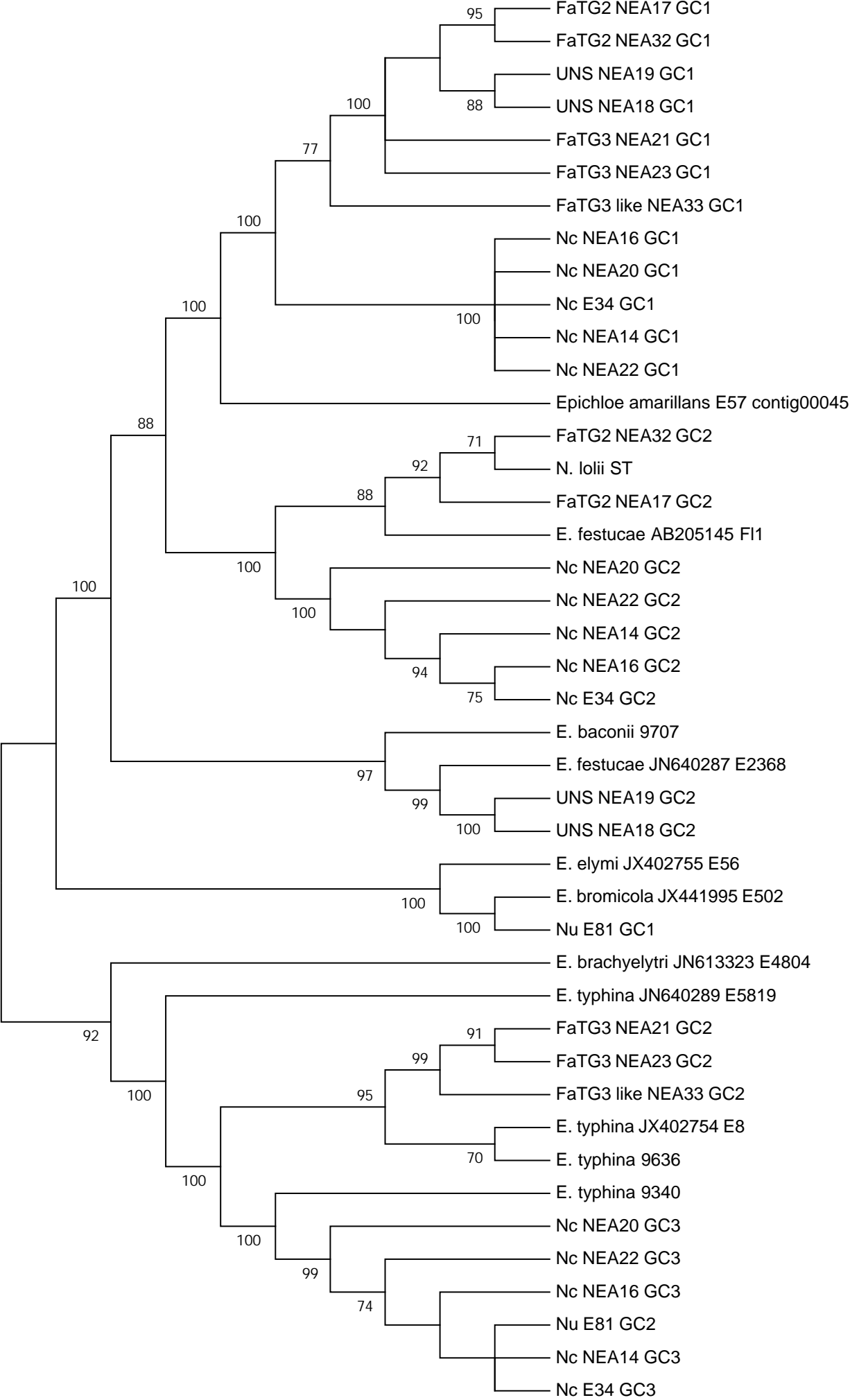

Supplement: Additional file 5 — Bootstrap consensus tree generated through maximum likelihood analysis of perA gene sequence among reference endophyte isolates and selected fescue endophytes. Branches with bootstrap values of greater than 70% from 1000 bootstrap replication are marked next to each branch. [file 1471-2148-13-270-S5.pdf]
