# Supplementary material for: Phylogenomics of fescue grass-derived fungal endophytes based on selected nuclear genes and the mitochondrial gene complement
Source: BMC Evol Biol. 2013 Dec 12;13:270. doi: 10.1186/1471-2148-13-270 (PMC4028799; doi:10.1186/1471-2148-13-270)
Supplement: Additional file 6 — Genbank accession numbers of the sequences represented in the phylogenetic study. [file 1471-2148-13-270-S6.docx]

**Additional File 6:** GenBank accession numbers of the sequences represented in the phylogenetic study

| **Taxonomic group** | **Isolate** | **Accession number** | | |
| --- | --- | --- | --- | --- |
|  |  | ***tefA*** | ***tub2*** | ***perA*** |
| *N. coenophialum* | NEA20 | KF811565 | KF811597 | KF811525 |
|  |  | KF811563 | KF811596 | KF811510 |
|  |  | KF811564 | KF811598 | KF811509 |
|  | NEA16 | KF811558 | KF811588 | KF811524 |
|  |  | KF811556 | KF811587 | KF811508 |
|  |  | KF811557 | KF811589 | KF811507 |
|  | NEA22 | KF811568 | KF811600 | KF811528 |
|  |  | KF811566 | KF811599 | KF811516 |
|  |  | KF811567 | KF811601 | KF811515 |
|  | NEA14 | KF811555 | KF811585 | KF811526 |
|  |  | KF811553 | KF811584 | KF811512 |
|  |  | KF811554 | KF811586 | KF811511 |
|  | E34 | KF811550 | KF811581 | KF811527 |
|  |  | KF811548 | KF811580 | KF811514 |
|  |  | KF811549 | KF811582 | KF811513 |
| *Fa*TG-2 | NEA17 | KF811559 | KF811590 | KF811532 |
|  |  | KF811560 | KF811591 | KF811521 |
|  | NEA32 | KF811543 | KF811575 | KF811533 |
|  |  | KF811544 | KF811576 | KF811520 |
| *Fa*TG-3 | NEA21 | KF811540 | KF811571 | KF811534 |
|  |  | KF811539 | KF811602 | KF811518 |
|  | NEA23 | KF811570 | KF811572 | KF811535 |
|  |  | KF811569 | KF811603 | KF811517 |
| *Fa*TG-3-like | NEA33 | KF811542 | KF811574 | KF811536 |
|  |  | KF811541 | KF811573 | KF811519 |
| UNS | NEA18 | KF811562 | KF811592 | KF811531 |
|  |  | KF811561 | KF811593 | KF811523 |
|  | NEA19 | KF811538 | KF811594 | KF811530 |
|  |  | KF811537 | KF811595 | KF811522 |
| *N. uncinatum* | E81 | KF811552 | KF811583 | KF811529 |
|  |  | KF811551 |  | KF811503 |
| *E. typhina* | 9340 | KF811545 | KF811577 | KF811504 |
| *E. typhina* | 9636 | KF811546 | KF811578 | KF811505 |
| *E. baconii* | 9707 | KF811547 | KF811579 | KF811506 |
