# Supplementary material for: Phylogenomics of fescue grass-derived fungal endophytes based on selected nuclear genes and the mitochondrial gene complement
Source: BMC Evol Biol. 2013 Dec 12;13:270. doi: 10.1186/1471-2148-13-270 (PMC4028799; doi:10.1186/1471-2148-13-270)
Supplement: Additional file 7 — Sequence output and de novo assembly statistics for sequenced fescue-derived endophytes. [file 1471-2148-13-270-S7.docx]

Additional File 7: Sequence output and *de novo* assembly statistics for sequenced fescue-derived endophytes

| Taxon | Strain/isolate ID | Yield (Mbases) | Number of Reads (millions) | Number of contigs | Number of bases (bp) | N50 contig size (bp) | Largest contig size (bp) |
| --- | --- | --- | --- | --- | --- | --- | --- |
| *E. typhina* | 9340 | 9493 | 104.5 | 9,599 | 28,359,241 | 54,864 | 254,366 |
| *E. typhina* | 9636 | 3523 | 36.5 | 18,451 | 29,751,940 | 22 937 | 210,783 |
| *E. baconii* | 9707 | 6480 | 73.5 | 23,413 | 30,726,298 | 21,781 | 152,824 |
| *N. coenophialum* | E34 | 7907 | 91.3 | 677,512 | 62,653,883 | 1,022 | 25,595 |
| *N. coenophialum* | NEA14 | 4063 | 43.5 | 458,842 | 44,701,782 | 472 | 8,992 |
| *N. coenophialum* | NEA16 | 2615 | 27.4 | 675,717 | 62,860,375 | 800 | 25,740 |
| *N. coenophialum* | NEA20 | 2722 | 28.6 | 526,879 | 75,594,133 | 1,147 | 24,193 |
| *N. coenophialum* | NEA22 | 8433 | 96.2 | 454,002 | 57,327,278 | 789 | 13,713 |
| *N. uncinatum* | E81 | 2126 | 24.3 | 405,912 | 48,678,033 | 2,707 | 96,978 |
| UNS | NEA18 | 3405 | 35.8 | 415,464 | 61,553,077 | 1,544 | 36,082 |
| UNS | NEA19 | 8217 | 93.9 | 656,200 | 51,168,954 | 1,192 | 30,947 |
| *Fa*TG-3 | NEA21 | 4515 | 47.6 | 487,598 | 51,088,371 | 1,555 | 49,106 |
| *Fa*TG-3 | NEA23 | 4321 | 46.5 | 599,435 | 54,621,344 | 5,096 | 62,665 |
| *Fa*TG-3-like | NEA33 | 2959 | 31.0 | 691,885 | 55,968,231 | 1,829 | 31,477 |
| *Fa*TG-2 | NEA32 | 2623 | 27.4 | 706,383 | 45,001,009 | 741 | 35,511 |
| *Fa*TG-2 | NEA17 | 4471 | 46.7 | 615,802 | 52,907,827 | 911 | 22,441 |
